# 2,6-Dichloro­phen­yl 4-chloro­benzoate

**DOI:** 10.1107/S1600536812047204

**Published:** 2012-11-24

**Authors:** M. M. M Abdoh, V. Srinivasa Murthy, B. C. Manjunath, S. Shashikanth, N. K. Lokanath

**Affiliations:** a Department of Physics, Faculty of Science, An Najah National University, Nabtus West Bank, Palestine; b Department of Studies in Chemistry, University of Mysore, Manasagangotri, Mysore 570 006, India; c Department of Studies in Physics, Manasagangotri, University of Mysore, Mysore, 570 006, India

## Abstract

In the title compound, C_13_H_7_Cl_3_O_2_, the dihedral angle between the benzene rings is 82.1 (2)°. The dihedral angle between the CO_2_ group and its carbon-bonded ring is 14.50 (19)° In the crystal, aromatic π–π stacking inter­actions [minimum ring centroid separation = 3.604 (2) Å] occur.

## Related literature

For background to benzophenones, see: Khanum *et al.* (2004 ▶, 2009 ▶). For a related structure, see: Gowda *et al.* (2008 ▶).
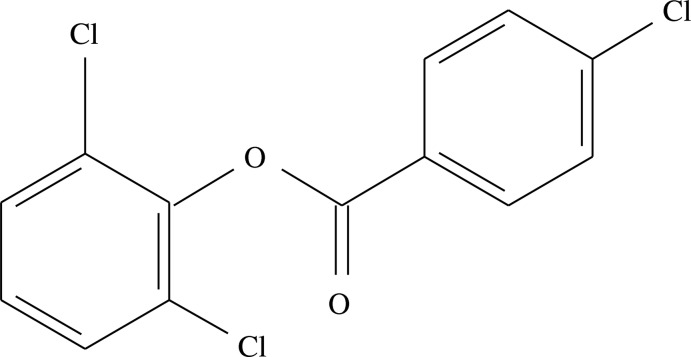


## Experimental

### 

#### Crystal data

C_13_H_7_Cl_3_O_2_
*M* *_r_* = 301.54
Triclinic, 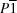

*a* = 7.1584 (10) Å
*b* = 8.1183 (13) Å
*c* = 11.5338 (16) Å
α = 95.352 (11)°
β = 99.852 (10)°
γ = 105.854 (10)°
*V* = 628.30 (17) Å^3^
*Z* = 2
Mo *K*α radiation
μ = 0.72 mm^−1^
*T* = 103 K
0.32 × 0.20 × 0.18 mm

#### Data collection

Oxford Diffraction Xcalibur CCD diffractometer
8510 measured reflections
2278 independent reflections
1738 reflections with *I* > 2σ(*I*)
*R*_int_ = 0.045

#### Refinement

*R*[*F* ^2^ > 2σ(*F* ^2^)] = 0.054
*wR*(*F* ^2^) = 0.158
*S* = 1.08
2278 reflections
163 parameters
H-atom parameters constrained
Δρ_max_ = 0.84 e Å^−3^
Δρ_min_ = −0.60 e Å^−3^

### 

Data collection: *CrysAlis PRO* (Oxford Diffraction, 2009 ▶); cell refinement: *CrysAlis PRO*; data reduction: *CrysAlis PRO* ; program(s) used to solve structure: *SHELXS97* (Sheldrick, 2008 ▶); program(s) used to refine structure: *SHELXL97* (Sheldrick, 2008 ▶); molecular graphics: *Mercury* (Macrae *et al.*, 2006 ▶); software used to prepare material for publication: *Mercury*.

## Supplementary Material

Crystal structure: contains datablock(s) global, I. DOI: 10.1107/S1600536812047204/hb6981sup1.cif

Structure factors: contains datablock(s) I. DOI: 10.1107/S1600536812047204/hb6981Isup2.hkl

Supplementary material file. DOI: 10.1107/S1600536812047204/hb6981Isup3.cml

Additional supplementary materials:  crystallographic information; 3D view; checkCIF report

## Figures and Tables

**Fig. 1.**
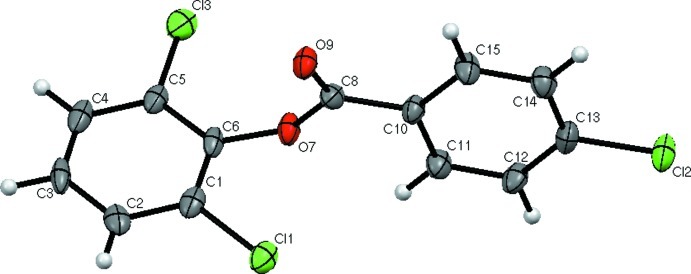
ORTEP diagram of the title compound showing 50% probability ellipsoids.

**Fig. 2.**
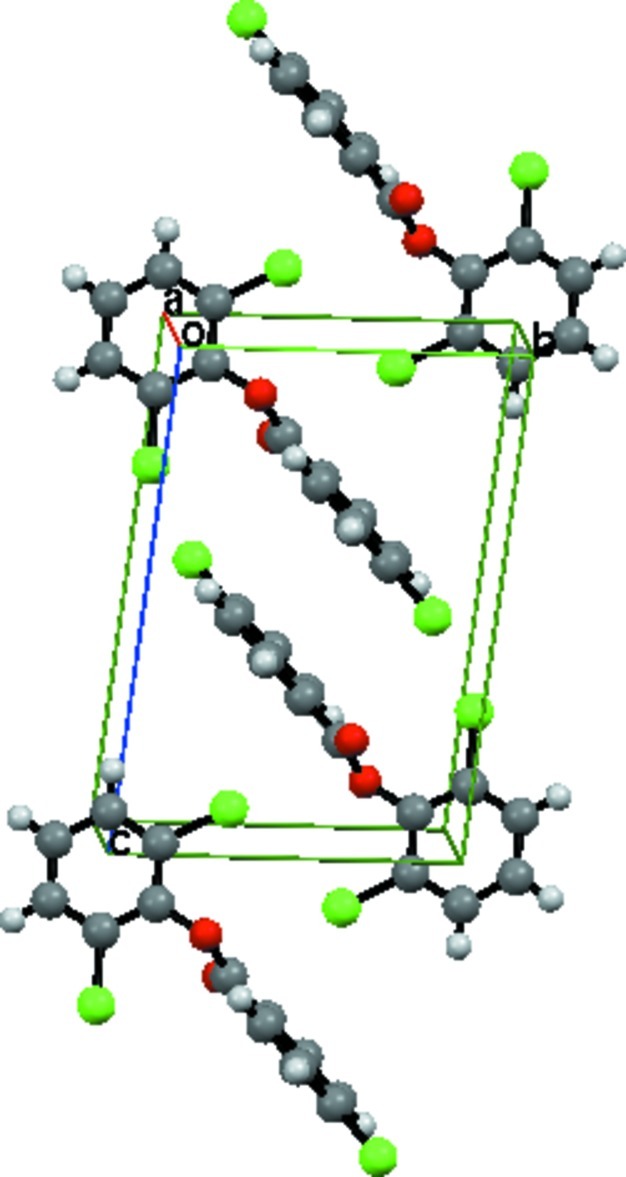
Packing diagram of the title compound, viewed along the crystallographic a axis.

## supplementary crystallographic information

### Comment

The benzophenone analogues find a unique place in medicinal chemistry and play a significant role with various pharmacological properties (Khanum *et al.*, 2004). In addition, they are reported to possess antifungal activity (Khanum *et al.*, 2009).

In the title molecule, C_13_H_7_Cl_3_O_2_ (Fig. 1.), dihedral angle between the terminal benzene rings bridged by corboxylate group is 82.1 (2) °, with the conformation of the chlorobenzene ring influenced by the presence of an intramolecular C11—H···O7 interaction [2.715 (4) Å]. The overall geometry of the title compound is similar to 2,6-dichlorophenyl 4-methylbenzoate (Gowda *et al.*, 2008).

The crystal structure (Fig. 2.) features *π*···*π* and C—Cl···*π* interactions. The distance between *Cg*(1): C1/C2/C3/C4/C5/C6 and *Cg*(1) is 3.604 (2) Å [-*x* + 1,-*y*,-*z* + 2] and 3.645 (2) Å [-*x*, -*y*, -*z* + 2].

### Experimental

To a stirred mixture of 2,6-dichlorophenol (1 g, 6.13 m*M*) and 4-chlorobenzoyl chloride (0.96 g, 5.52 m*M*, 0.9 eq), 20 ml of 10% aqueous sodium hydroxide was added dropwise at room temperature. The reaction mass was stirred for 1 h. The separated solid was filtered and dissolved in 2 ml diethyl ether. The organic layer was washed with water (3 × 15 ml) and dried over anhydrous sodium sulfate. The solvent was removed under reduced pressure to afford 2, 6-dichlorophenyl-4- chrolobenzoate (1.52 g, 82%, *M*. P = 98°C) as a white solid, which was recrystallized as colourless blocks using ethyl alcohol.

IR: 1760 cm^-1^(COO). ^1^H NMR:600Mhz (CDCl_3_) *δ* 7.17–7.21(1*H*,t), 7.39–7.41 (2*H*,d), 7.41–7.51 (2*H*,d), 8.18–8.20 (2*H*,d)

### Refinement

All hydrogen atoms were located geometrically with C—H = 0.93–0.97) Å and allowed to ride on their parent atoms with *U*_iso_(H) = 1.2*U*_eq_(aromatic C).

### Crystal data

**Table:**

C_13_H_7_Cl_3_O_2_	*Z* = 2
*M**_r_* = 301.54	*F*(000) = 304
Triclinic, *P*1	*D*_x_ = 1.594 Mg m^−^^3^
Hall symbol: -P 1	Melting point: 371 K
*a* = 7.1584 (10) Å	Mo *K*α radiation, λ = 0.71073 Å
*b* = 8.1183 (13) Å	Cell parameters from 2278 reflections
*c* = 11.5338 (16) Å	θ = 1.8–26.0°
α = 95.352 (11)°	µ = 0.72 mm^−^^1^
β = 99.852 (10)°	*T* = 103 K
γ = 105.854 (10)°	Block, colourless
*V* = 628.30 (17) Å^3^	0.32 × 0.20 × 0.18 mm

### Data collection

**Table:**

Oxford Diffraction Xcalibur CCD diffractometer	1738 reflections with *I* > 2σ(*I*)
Radiation source: fine-focus sealed tube	*R*_int_ = 0.045
Graphite monochromator	θ_max_ = 26.0°, θ_min_ = 1.8°
Detector resolution: 16.0839 pixels mm^-1^	*h* = −8→8
ω scans	*k* = −10→10
8510 measured reflections	*l* = −14→14
2278 independent reflections	

### Refinement

**Table:**

Refinement on *F*^2^	Primary atom site location: structure-invariant direct methods
Least-squares matrix: full	Secondary atom site location: difference Fourier map
*R*[*F*^2^ > 2σ(*F*^2^)] = 0.054	Hydrogen site location: inferred from neighbouring sites
*wR*(*F*^2^) = 0.158	H-atom parameters constrained
*S* = 1.08	*w* = 1/[σ^2^(*F*_o_^2^) + (0.0961*P*)^2^ + 0.2256*P*] where *P* = (*F*_o_^2^ + 2*F*_c_^2^)/3
2278 reflections	(Δ/σ)_max_ = 0.001
163 parameters	Δρ_max_ = 0.84 e Å^−^^3^
0 restraints	Δρ_min_ = −0.60 e Å^−^^3^

### Special details

**Table:**

Geometry. Bond distances, angles *etc*. have been calculated using the rounded fractional coordinates. All su's are estimated from the variances of the (full) variance-covariance matrix. The cell e.s.d.'s are taken into account in the estimation of distances, angles and torsion angles
Refinement. Refinement on *F*^2^ for ALL reflections except those flagged by the user for potential systematic errors. Weighted *R*-factors *wR* and all goodnesses of fit *S* are based on *F*^2^, conventional *R*-factors *R* are based on *F*, with *F* set to zero for negative *F*^2^. The observed criterion of *F*^2^ > σ(*F*^2^) is used only for calculating -*R*-factor-obs *etc*. and is not relevant to the choice of reflections for refinement. *R*-factors based on *F*^2^ are statistically about twice as large as those based on *F*, and *R*-factors based on ALL data will be even larger.

### Fractional atomic coordinates and isotropic or equivalent isotropic displacement parameters (Å^2^)

**Table:**

	*x*	*y*	*z*	*U*_iso_*/*U*_eq_	
Cl1	0.32525 (12)	−0.00436 (13)	0.72254 (8)	0.0296 (3)	
Cl2	0.35105 (14)	−0.85956 (14)	0.44115 (9)	0.0354 (3)	
Cl3	0.19259 (12)	−0.30913 (13)	1.10648 (8)	0.0322 (3)	
O7	0.2686 (3)	−0.2891 (3)	0.8624 (2)	0.0248 (8)	
O9	−0.0500 (3)	−0.3628 (4)	0.7612 (2)	0.0271 (8)	
C1	0.2821 (5)	0.0096 (5)	0.8663 (3)	0.0248 (10)	
C2	0.2782 (5)	0.1655 (5)	0.9231 (3)	0.0269 (11)	
C3	0.2488 (5)	0.1744 (6)	1.0399 (3)	0.0289 (11)	
C4	0.2242 (5)	0.0298 (5)	1.0965 (3)	0.0290 (13)	
C5	0.2260 (4)	−0.1256 (5)	1.0378 (3)	0.0235 (10)	
C6	0.2533 (4)	−0.1378 (5)	0.9200 (3)	0.0224 (10)	
C8	0.1124 (5)	−0.3822 (5)	0.7724 (3)	0.0223 (10)	
C10	0.1749 (5)	−0.5020 (5)	0.6946 (3)	0.0227 (10)	
C11	0.3746 (5)	−0.4855 (5)	0.6964 (3)	0.0264 (11)	
C12	0.4286 (5)	−0.5932 (5)	0.6183 (3)	0.0283 (11)	
C13	0.2838 (5)	−0.7194 (5)	0.5378 (3)	0.0272 (11)	
C14	0.0830 (5)	−0.7394 (5)	0.5340 (3)	0.0272 (11)	
C15	0.0296 (5)	−0.6312 (5)	0.6119 (3)	0.0281 (11)	
H2	0.29510	0.26470	0.88370	0.0320*	
H3	0.24570	0.28060	1.08060	0.0350*	
H4	0.20590	0.03730	1.17630	0.0350*	
H11	0.47420	−0.39890	0.75230	0.0320*	
H12	0.56480	−0.58090	0.61970	0.0340*	
H14	−0.01560	−0.82690	0.47810	0.0330*	
H15	−0.10690	−0.64390	0.61000	0.0340*	

### Atomic displacement parameters (Å^2^)

**Table:**

	*U*^11^	*U*^22^	*U*^33^	*U*^12^	*U*^13^	*U*^23^
Cl1	0.0248 (5)	0.0392 (6)	0.0206 (5)	0.0065 (4)	0.0017 (3)	−0.0027 (4)
Cl2	0.0410 (6)	0.0383 (7)	0.0287 (5)	0.0173 (4)	0.0097 (4)	−0.0077 (5)
Cl3	0.0255 (5)	0.0408 (7)	0.0250 (5)	0.0041 (4)	0.0029 (4)	−0.0004 (5)
O7	0.0182 (12)	0.0304 (16)	0.0218 (12)	0.0080 (10)	−0.0010 (9)	−0.0090 (12)
O9	0.0186 (12)	0.0338 (17)	0.0259 (13)	0.0086 (10)	0.0015 (10)	−0.0080 (13)
C1	0.0126 (16)	0.038 (2)	0.0202 (17)	0.0080 (14)	−0.0015 (13)	−0.0065 (17)
C2	0.0133 (16)	0.028 (2)	0.034 (2)	0.0049 (14)	−0.0012 (14)	−0.0074 (19)
C3	0.0155 (17)	0.034 (2)	0.030 (2)	0.0072 (14)	−0.0022 (14)	−0.0186 (18)
C4	0.0131 (16)	0.046 (3)	0.0215 (18)	0.0067 (15)	−0.0005 (13)	−0.0130 (19)
C5	0.0130 (16)	0.035 (2)	0.0175 (17)	0.0040 (14)	0.0001 (12)	−0.0064 (17)
C6	0.0125 (15)	0.030 (2)	0.0190 (17)	0.0049 (13)	−0.0020 (12)	−0.0112 (17)
C8	0.0175 (17)	0.025 (2)	0.0192 (17)	0.0012 (14)	0.0013 (13)	−0.0029 (16)
C10	0.0191 (17)	0.028 (2)	0.0186 (17)	0.0066 (14)	0.0022 (13)	−0.0046 (17)
C11	0.0200 (17)	0.030 (2)	0.0233 (18)	0.0033 (14)	0.0006 (14)	−0.0059 (17)
C12	0.0182 (17)	0.039 (2)	0.0259 (19)	0.0082 (15)	0.0051 (14)	−0.0045 (18)
C13	0.033 (2)	0.028 (2)	0.0235 (19)	0.0140 (16)	0.0087 (15)	−0.0016 (18)
C14	0.0249 (18)	0.029 (2)	0.0235 (19)	0.0070 (15)	−0.0008 (14)	−0.0038 (18)
C15	0.0173 (17)	0.032 (2)	0.029 (2)	0.0055 (15)	−0.0012 (14)	−0.0094 (19)

### Geometric parameters (Å, º)

**Table:**

Cl1—C1	1.737 (4)	C10—C11	1.395 (5)
Cl2—C13	1.737 (4)	C10—C15	1.403 (5)
Cl3—C5	1.731 (4)	C11—C12	1.372 (5)
O7—C6	1.381 (4)	C12—C13	1.378 (5)
O7—C8	1.376 (4)	C13—C14	1.394 (5)
O9—C8	1.202 (4)	C14—C15	1.371 (5)
C1—C2	1.379 (5)	C2—H2	0.9500
C1—C6	1.380 (5)	C3—H3	0.9500
C2—C3	1.397 (5)	C4—H4	0.9500
C3—C4	1.380 (6)	C11—H11	0.9500
C4—C5	1.379 (5)	C12—H12	0.9500
C5—C6	1.404 (5)	C14—H14	0.9500
C8—C10	1.471 (5)	C15—H15	0.9500
			
C6—O7—C8	117.2 (3)	C11—C12—C13	119.5 (4)
Cl1—C1—C2	119.7 (3)	Cl2—C13—C12	119.8 (3)
Cl1—C1—C6	118.1 (3)	Cl2—C13—C14	119.1 (3)
C2—C1—C6	122.2 (3)	C12—C13—C14	121.2 (3)
C1—C2—C3	118.5 (4)	C13—C14—C15	119.1 (3)
C2—C3—C4	120.4 (4)	C10—C15—C14	120.5 (3)
C3—C4—C5	120.3 (3)	C1—C2—H2	121.00
Cl3—C5—C4	121.1 (3)	C3—C2—H2	121.00
Cl3—C5—C6	118.7 (3)	C2—C3—H3	120.00
C4—C5—C6	120.2 (3)	C4—C3—H3	120.00
O7—C6—C1	120.4 (3)	C3—C4—H4	120.00
O7—C6—C5	121.1 (3)	C5—C4—H4	120.00
C1—C6—C5	118.3 (3)	C10—C11—H11	120.00
O7—C8—O9	122.9 (3)	C12—C11—H11	120.00
O7—C8—C10	110.6 (3)	C11—C12—H12	120.00
O9—C8—C10	126.5 (3)	C13—C12—H12	120.00
C8—C10—C11	121.9 (3)	C13—C14—H14	120.00
C8—C10—C15	119.0 (3)	C15—C14—H14	120.00
C11—C10—C15	119.0 (3)	C10—C15—H15	120.00
C10—C11—C12	120.7 (3)	C14—C15—H15	120.00
			
C8—O7—C6—C1	−76.3 (4)	C4—C5—C6—O7	175.7 (3)
C8—O7—C6—C5	109.5 (4)	C4—C5—C6—C1	1.3 (5)
C6—O7—C8—O9	−18.4 (5)	O7—C8—C10—C11	−15.5 (5)
C6—O7—C8—C10	160.7 (3)	O7—C8—C10—C15	167.8 (3)
Cl1—C1—C2—C3	−178.2 (3)	O9—C8—C10—C11	163.6 (4)
C6—C1—C2—C3	1.6 (6)	O9—C8—C10—C15	−13.1 (6)
Cl1—C1—C6—O7	3.2 (5)	C8—C10—C11—C12	−176.4 (3)
Cl1—C1—C6—C5	177.6 (3)	C15—C10—C11—C12	0.3 (5)
C2—C1—C6—O7	−176.6 (3)	C8—C10—C15—C14	176.7 (3)
C2—C1—C6—C5	−2.2 (5)	C11—C10—C15—C14	−0.1 (6)
C1—C2—C3—C4	−0.1 (6)	C10—C11—C12—C13	−0.4 (6)
C2—C3—C4—C5	−0.8 (6)	C11—C12—C13—Cl2	−178.4 (3)
C3—C4—C5—Cl3	−179.3 (3)	C11—C12—C13—C14	0.2 (6)
C3—C4—C5—C6	0.1 (5)	Cl2—C13—C14—C15	178.7 (3)
Cl3—C5—C6—O7	−4.9 (4)	C12—C13—C14—C15	0.0 (6)
Cl3—C5—C6—C1	−179.3 (3)	C13—C14—C15—C10	−0.1 (6)
